# Oxidative Stress Markers to Investigate the Effects of Hyperoxia in Anesthesia

**DOI:** 10.3390/ijms20215492

**Published:** 2019-11-04

**Authors:** Sara Ottolenghi, Federico Maria Rubino, Giovanni Sabbatini, Silvia Coppola, Alice Veronese, Davide Chiumello, Rita Paroni

**Affiliations:** 1 Dipartimento di Scienze della Salute, Università degli Studi di Milano, 20142 Milan, Italy; federico.rubino@unimi.it (F.M.R.); davide.chiumello@unimi.it (D.C.) rita.paroni@unimi.it (R.P.); 2 SC Anestesia e Rianimazione, Ospedale San Paolo—Polo Universitario, ASST Santi Paolo e Carlo; 20142 Milan, Italy; giovanni.sabbatini1@gmail.com (G.S.); silvia_coppola@libero.it (S.C.); alice.veronese@unimi.it (A.V.); 3 Coordinated Research Center on Respiratory Failure, University of Milan, 20123 Milan, Italy

**Keywords:** hyperoxia, oxygen, oxidative stress, malondialdehyde, glutathionyl hemoglobin, hypoxia, anesthesia, biomarkers, hydroperoxides, nitric oxide

## Abstract

Oxygen (O_2_) is commonly used in clinical practice to prevent or treat hypoxia, but if used in excess (hyperoxia), it may act as toxic. O_2_ toxicity arises from the enhanced formation of Reactive Oxygen Species (ROS) that exceed the antioxidant defenses and generate oxidative stress. In this study, we aimed at assessing whether an elevated fraction of inspired oxygen (FiO_2_) during and after general anesthesia may contribute to the unbalancing of the pro-oxidant/antioxidant equilibrium. We measured five oxidative stress biomarkers in blood samples from patients undergoing elective abdominal surgery, randomly assigned to FiO_2_ = 0.40 vs. 0.80: hydroperoxides, antioxidants, nitrates and nitrites (NO_x_), malondialdehyde (MDA), and glutathionyl hemoglobin (HbSSG). The MDA concentration was significantly higher 24 h after surgery, and the body antioxidant defense lower, in the FiO_2_ = 0.80 group with respect to both the FiO_2_ = 0.40 group and the baseline values (*p* ≤ 0.05, Student’s *t*-test). HbSSG in red blood cells was also higher in the FiO_2_ = 0.80 group at the end of the surgery. NO_x_ was higher in the FiO_2_ = 0.80 group than the FiO_2_ = 0.40 group at t = 2 h after surgery. MDA, the main end product of the peroxidation of polyunsaturated fatty acids directly influenced by FiO_2_, may represent the best marker to assess the pro-oxidant/antioxidant equilibrium after surgery.

## 1. Introduction

Oxygen (O_2_) is commonly administered during general anesthesia to avoid the risk of tissue hypoxia. On the other hand, O_2_ excess constitutes a patho-physiological condition defined as hyperoxia. Our organism responds to hyperoxia by adaptive and/or potentially harmful mechanisms surprisingly similar to the ones involved in the adaptation to hypoxia [1,2].

**Figure 1:**
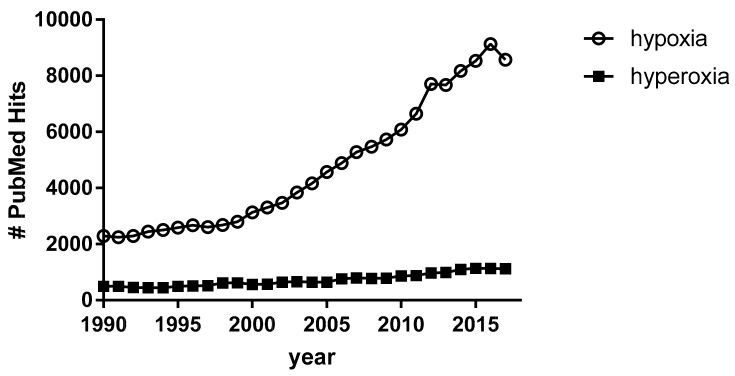
PubMed hits per year for “hypoxia” vs. hits for “hyperoxia”.

Hypoxia deserves major consideration because it represents a clinical condition afflicting millions of people worldwide and constitutes an important source of social and economic distress [3,4]. Hypoxia has developed a growing interest throughout the years, as demonstrated by the number of PubMed hits, which increased from 2285 articles in 1990 to 8528 in 2015, i.e., a nearly four-fold increase. Differently, hyperoxia has gained much less consideration. PubMed hits for the term “hyperoxia”, despite a slight linear increase over the years, represent one-tenth of those related to the term “hypoxia” (Figure 1), although hyperoxia is not free from deleterious effects [5,6]. The two conditions lie at the opposite sides of the hormetic curve [7] of physiological adaptation to variations in the availability of O_2_ [2,8,9].

Despite the unquestionable utility of O_2_ administration in the case of hypoxia, there is conflicting evidence regarding the benefit/risk balance of the high perioperative fraction of inspired oxygen (FiO_2_) in previously non-hypoxic subjects as a preventive measure [10]. On the one hand, the PROXI trial [11], comparing patients undergoing acute or elective laparotomy receiving FiO_2_ = 0.80 versus 0.40, resulted in no difference in the incidence of surgical site infections in 1400 patients. On the other hand, both a meta-analysis [12] and the WHO recommendations suggested a perioperative and intraoperative FiO_2_ administration of 0.80 to prevent surgical site infection [13].

Moreover, hyperoxia has also been shown to be per se an independent mortality risk factor in Intensive Care Unit patients [14]. Several studies have demonstrated that it may induce damage in the lungs [15,16] and brain [17], both in vivo (animal models) and in vitro, through the excessive production of Reactive Oxygen Species (ROS) and the consequently unbalanced oxidative stress.

Nitric oxide (NO) seems to play a pivotal role in this mechanism because its level is strongly affected by the levels of circulating ROS, thereby altering the vascular relaxation through the guanosine 3′,5′-cyclic monophosphate (cGMP) mechanism [18].

Despite several ongoing investigations [19], a clear role for high FiO_2_ in triggering oxidative stress through ROS generation in patients undergoing surgery has not yet been clarified. In addition, optimal methods to predict the benefit/risk balance of hyperoxia in such patients still need to be identified.

The aim of this study is to evaluate the effect of high perioperative FiO_2_ on the redox equilibrium in terms of the blood levels of oxidative stress and antioxidant response markers in patients undergoing elective abdominal surgery.

## 2. Results

### 2.1. Study Population

We recruited 25 patients. Of these, five were excluded from clinical and biochemical analysis for the following reasons: two were hospitalized in the Intensive Care Unit after surgery because of comorbidities; one required blood transfusion during surgery; and two underwent surgery procedures with operative times <90 min. Therefore, 20 patients (10 per group) were considered for analysis. Most of the recruited patients underwent laparoscopic hemicolectomy. Two patients (one per group) required the conversion to open surgery after the first time point, because of anatomical reasons. Two more patients (one per group) underwent a hernia repair laparoscopic surgery. Among all of them, 12 (6 + 6, well balanced for surgery type between the two groups) were assayed for oxidative stress markers. All patients were evaluated in the anesthetic clinic in advance. According to the local guidelines, preoperative fasting was prescribed (6 h for solid food and 2 h for clear fluids prior to surgery). Patients received premedication before the induction of general anesthesia. The subjects were monitored with three leads electrocardiogram, peripheral oxygen saturation, either invasive or intermittent noninvasive blood pressure, esophageal temperature, end-tidal CO_2_, and neuro-muscular blockade monitoring. A bladder catheter was inserted for urine output monitoring. General anesthesia was induced with Propofol, Fentanest, and Rocuronium. After tracheal intubation, patients were mechanically ventilated and general anesthesia was maintained with a volatile agent, either Sevoflurane (one patient per group) or Desflurane, and Remifentanil infusion. Postoperative analgesia was provided by the parenteral administration of morphine and nonsteroidal anti-inflammatory drugs. Epidural anesthesia was performed in four patients (two per group). None of the considered patients underwent significant blood loss (>500 mL) during surgery. Table 1 shows the demographics and anthropometric characteristics of the study population. There was no significant difference between the two groups. None of the investigated patients developed early postoperative complications (in terms of an infarct, electrocardiographic alterations, peripheral desaturation requiring further O_2_ treatment, or wound infection) in the first 24 h after surgery.

### 2.2. Blood Gas Analysis

**Figure 2:**
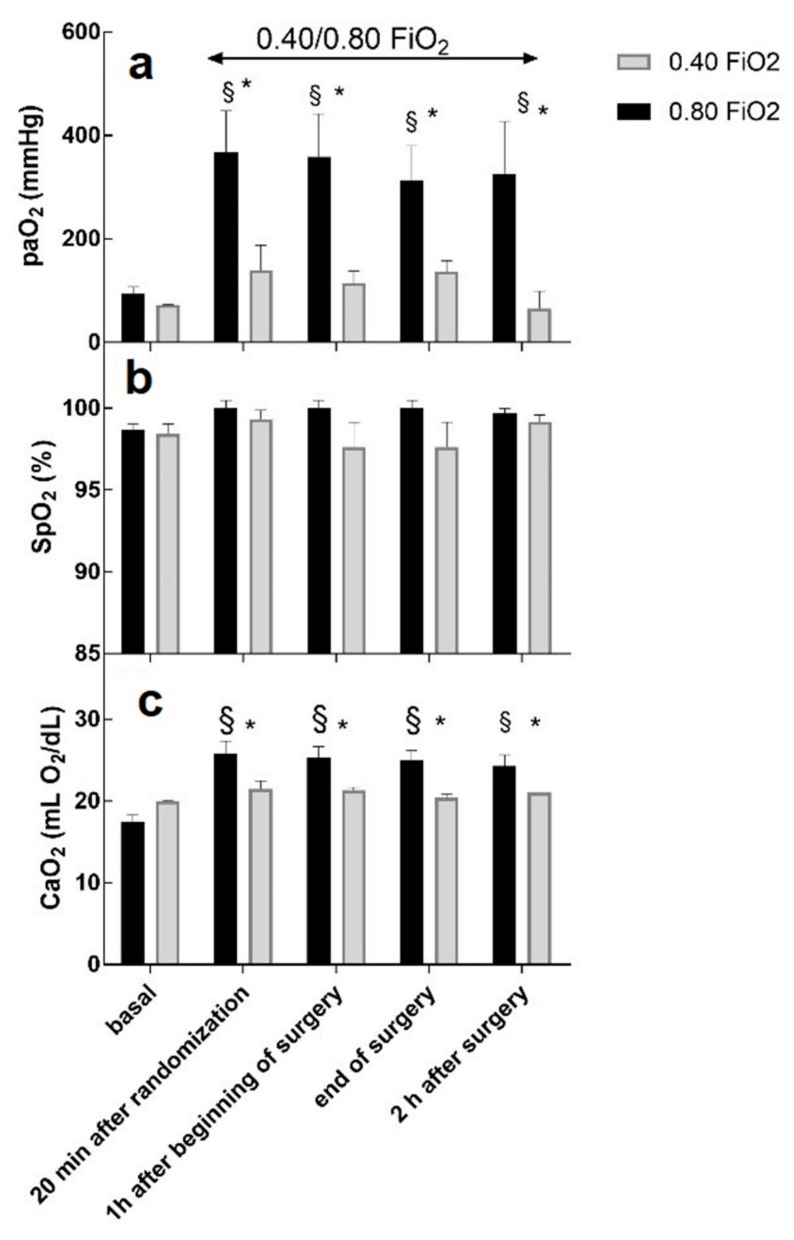
Time course of PaO_2_ (**a**), SpO_2_ (**b**), and CaO_2_ (**c**) during the experimental protocol. Data are expressed as mean ± standard error of the mean (SEM). § *p* < 0.05 when compared to basal value (two-way ANOVA test, Dunnett’s method); * *p* < 0.05 FiO_2_ = 0.40 vs. 0.80 (Student’s *t*-test).

Figure 2 shows the values of the arterial partial pressure of oxygen (PaO_2_), the peripheral saturation of O_2_ (SpO_2_), and the arterial concentration of oxygen (CaO_2_) at each timing in each group.

As expected, PaO_2_ and CaO_2_ were different between the groups at each timing. Compared to the basal timing, PaO_2_ and CaO_2_ were significantly higher in the FiO_2_ = 0.80 group.

No change was noticed in the SpO_2_ values, neither between the two groups nor compared to the basal values.

To obtain a more representative descriptor of the O_2_ dose, the FiO_2_ and surgery time were combined. This descriptor of O_2_ dose yielded a better appreciation of the time course of the selected markers of oxidative stress, as shown below.

### 2.3. Oxidative Stress Markers

**Figure 3:**
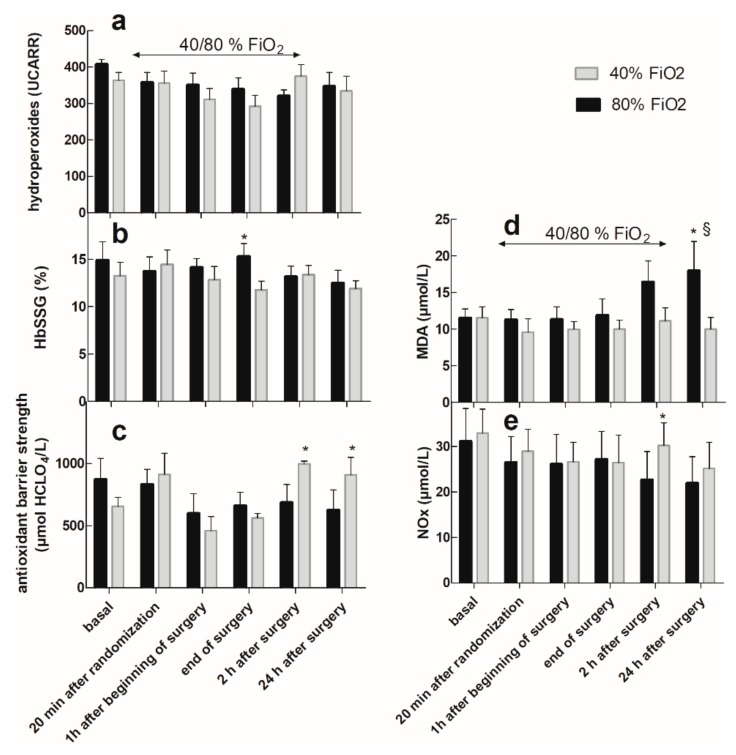
Oxidative stress markers: (**a**) hydroperoxides; (**b**) glutathionyl hemoglobin (HbSSG); (**c**) antioxidant barrier strength; (**d**) malondialdehyde (MDA); (**e**) Nitrites and nitrates (NO_x_). Data are expressed as mean ± SEM. § *p* < 0.05 when compared to basal value (two-way ANOVA test, Dunnett’s method); * *p* < 0.05 FiO_2_ = 0.40 vs. 0.80 (Student’s *t*-test).

Figure 3 reports the levels of the five measured oxidative stress biomarkers at the different time points of the study protocol.

Hydroperoxides, expressed as Carratelli Units (UCARR), (panel a), did not highlight any differences between the two groups nor within the same group, with respect to the basal value. Remarkably, the basal value of all patients was >300 UCARR, which is, according to the manufacturer’s instructions, the threshold value to define oxidative stress.

As shown in panel b, most patients showed a high, but constant, level of HbSSG, expressed as a percentage of the total β Hb chain, before and throughout the surgery, with little influence from the FiO_2_ level, except at the end of surgery, when they were higher in the FiO_2_ = 0.80 group. Overall values were higher than those reported in the literature for healthy subjects [20].

The strength of the antioxidant barrier, expressed as mmol HClO/L (panel c), was significantly higher at *t* = 2 h and *t* = 24 h after surgery in the FiO_2_ = 0.40 group compared with both the basal value and the corresponding value in the FiO_2_ = 0.80 group.

Conversely, MDA (panel d) started to be higher in the FiO_2_ = 0.80 group vs. the FiO_2_ = 0.40 group 2 h after surgery, although not yet significantly. The difference reached significance 24 h after the surgery with respect to both the basal value and the corresponding value in the FiO_2_ = 0.40 group. The level of total NO_x_ (panel e) was lower in the FiO_2_ = 0.80 group than in the FiO_2_ = 0.40 group at *t* = 2 h after surgery.

**Figure 4:**
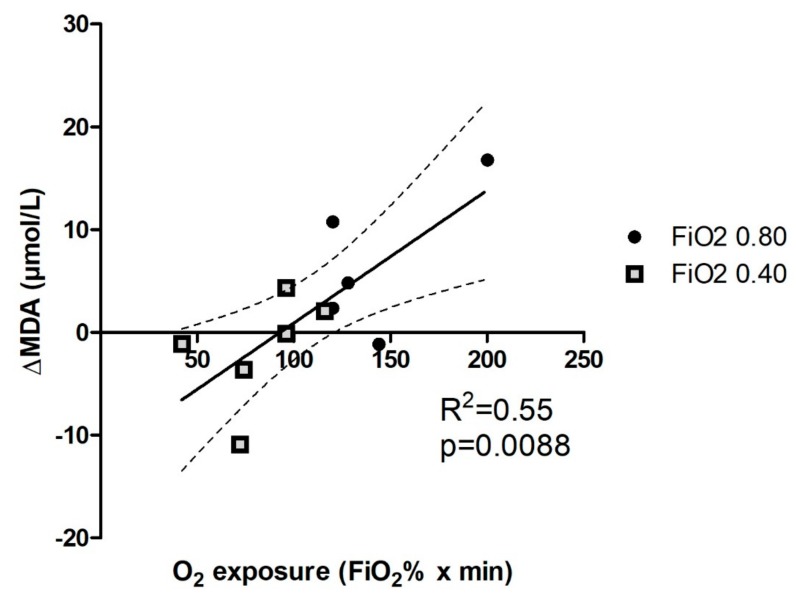
Scatter plot of MDA production at 24 h as function of the O_2_ exposure. O_2_ exposure was calculated as the product of FiO_2_ and the surgery running time. Solid line: linear regression line. Dotted lines: 95% confidence bands.

A better appreciation of the time course of MDA formation is displayed in Figure 4, where the net formation of MDA is expressed as the difference between pre-surgery levels and those measured 24 h after surgery.

The likely cause of MDA formation as a late product of lipid oxidative degradation is described as the O_2_ dose and proxied as the product of its inhaled fraction volume and time. The result of this elaboration at 24 h is displayed as the clearest example. Although the number of examined patients was small, due to the pilot character of this study, MDA formation showed a monotonically increasing trend with a statistically significant slope and a better than 50% regression coefficient. Four out of five patients in the 0.80 FiO_2_ group and only two out of six in the 0.40 FiO_2_ group showed an increased production of circulating MDA. One patient in the 0.80 FiO_2_ group and two in the 0.40 FiO_2_ group did not show a significant change from the basal value, and two patients in the 0.40 FiO_2_ group showed a decrease in MDA production. When the O_2_ dose was calculated taking into account not only actual surgery time but also post-surgery inhalation of the same enriched gas mixture through a non-mechanical device, and even the time that patients spent after surgery breathing ambient air, the strength of the correlation progressively fell towards non-significance.

## 3. Discussion

In this study, we found that the intraoperative and postoperative administration of FiO_2_ = 0.80 in anesthetized patients undergoing abdominal surgery has an impact on the redox equilibrium at 24 h after surgery, as witnessed by increased lipid peroxidation and decreased antioxidant barrier strength. With this study, we also contribute to a much felt need from anesthesiologists and surgeons to find reliable indicators of organism resilience to oxidative stress for the optimal management of anesthesia and post-surgery recovery.

Measured PaO_2_ was three times higher in the FiO_2_ = 0.80 group with respect to the FiO_2_ = 0.40 group. Similarly, calculated CaO_2_ was also higher in the FiO_2_ = 0.80 group with respect to the FiO_2_ = 0.40 group, thus providing support to the hypothesis that a greater systemic oxygenation in the FiO_2_ = 0.80 group may be responsible for potentially noxious oxidative stress.

PaO_2_ reflects the concentration of dissolved oxygen in arterial blood, whereas tissue oxygenation might depend mainly on the amount of oxygen bound to hemoglobin. Not unexpectedly, SpO_2_ was not significantly influenced by the two explored FiO_2_ values of the study protocol because all patients of the FiO_2_ = 0.40 group were already hyperoxic.

**Figure 5:**
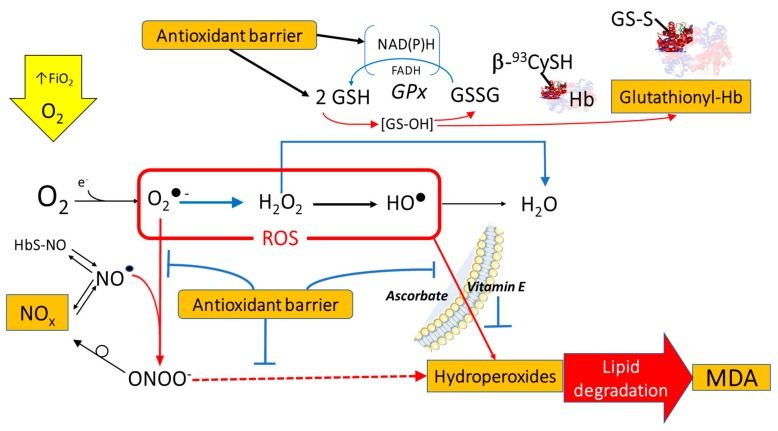
Some mechanisms for the production of the main Reactive Oxygen Species (ROS) from excess dissolved molecular oxygen (O_2_) and the biomarkers used to evaluate its effects. Orange squares: oxidative stress markers considered in this study. Blue arrows: reduction processes. Red arrows: oxidation processes.

As oxidative stress is the result of excess ROS and impaired anti-oxidant defense, we evaluated both these aspects, each with specific biomarkers, to assess the establishment or rupture of the pro-oxidant/antioxidant balance. The links between the chosen markers and the overall mechanisms of ROS production and toxicity are summarized in Figure 5. A description of the relationships between the examined biomarkers is reported in the Supplementary Materials document.

The plasma level of MDA, the main product of the lipid peroxidation of polyunsaturated fatty acid, assessed through the TBARS test, highlights oxidative stress as a late effect of hyperoxia. The role of antioxidant defenses for the high MDA level 24 h after surgery (Figure 3d) in the FiO_2_ = 0.80 group was demonstrated by the levels of antioxidant barrier strength that revealed decreased antioxidant defense in this group of patients (Figure 3c). It is thus intuitive to conclude that the higher level of lipid peroxidation in the FiO_2_ = 0.80 group is mainly attributable to the hyperoxia-induced consumption of the antioxidant barrier. This situation may end in oxidative damage to the plasma membranes. In this biochemical context, MDA and the antioxidant barrier strength can be considered as instruments to predict hyperoxia-related oxidative damage in surgery. Low antioxidant barrier strength, often associated with high values of hydroperoxides, has also been observed as a predictor of cardiovascular events in patients with coronary disease [21].

The plasma NO_x_ level may give information about the effect of oxidative stress on the modulation of the vasorelaxation by NO^•^. This reaction decreases the size of the NO^•^ pool. Thus, the observed decrease in NO_x_ at *t* = 2 h after surgery in the FiO_2_ = 0.80 group (Figure 3e) may be considered a cardiovascular risk factor [22] because it decreases vasodilatation and may impair myocardial tissue perfusion and oxygenation.

The main antioxidant mechanism in RBCs is H_2_O_2_ scavenging by reduced glutathione (GSH), with the final generation of HbSSG. In this study, most patients showed a constant level of HbSSG before and throughout the surgery. A similar behavior was observed in patients who underwent carotid surgery, characterized by constant low levels of HbSSG. [19]. On the other hand, our patients had high HbSSG levels even before surgery, which are consistent with the almost complete binding of RBC glutathione to hemoglobin (saturation). The observation of no further increase of HbSSG during surgery, independent from the high inhaled oxygen concentration, may just reflect this phenomenon. The RBC glutathione levels in the population varied within a factor of six (0.6–3.6 mmol/L) and the strength of its antioxidant barrier (measured as the redox potential E_hc_) showed the same distribution, thus suggesting that the overall trait of glutathione homeostasis is under individual genetic control [23]. This fact can in turn cooperate in accounting for the different starting levels of individual HbSSG fraction and for its resistance to change during the surgery.

Hydroperoxides are generated by the reaction of excess ROS with organic molecules. We were unable to detect significant variations of this parameter, and our failure is consistent with the literature where data was gathered in patients undergoing surgery at FiO_2_ = 0.50 [19]. According to that study, the antioxidant barrier may limit damage led by ROS. Here, we showed that the noxious effects of oxidative stress can be revealed by the more sensitive TBARS test, and that damage is measurable 24 h after surgery. High basal values of hydroperoxides (>300 UCARR, threshold value to assess a situation of oxidative stress [24]) are commonly found in aged (>69 years) healthy subjects [25]. These high values, together with the high basal values of HbSSG, may also be due to both the age and smoking habits of some of the patients involved in this study (20%). Preoperative hydroperoxides have been proposed as a factor indicating vulnerability to surgical oxidative stress [26]. As intermediate products of ROS degradation, hydroperoxides are further oxidized into MDA. This mechanism may explain the slight decrease of hydroperoxides (measured as dROMs) during surgery with no increase after surgery. MDA can also be considered as a chemoattractant for immunity cells [27], which may play a role in the previously reported [12,28] effects of O_2_ on wound healing. In addition, the production of MDA is best related to the intensity of exposure to hyperoxia during surgery, rather than to the total O_2_ dose during the entire observation time of the subjects. This observation, although still preliminary, suggests that MDA production is likely higher in conditions of exposure to a very high partial pressure of O_2_ concentration, even for a short time, rather than by longer exposure at lower partial pressure. Both shorter and longer surgeries should be examined to better understand the appropriateness of the simplified Haber’s concentration × time dose metrics equation [29] to rationalize the effects of exposure to high O_2_ levels [30].

### Limitations

Since most initial ROS are characterized by a very short half-life and cannot be measured directly, there are several biomarkers to assess oxidative stress, and each has a specific time frame and biological meaning. We chose the most frequently examined, as they are endowed with a clearer biological meaning. We may assume that a more comprehensive panel of biomarkers will improve our understanding of some key points in the oxidative stress cascade.

This study is a monocentric pilot study, due to the logistic requirement to centralize biochemical measurements, especially of the more short-lived biomarkers. A more extensive study on a wider choice of subjects and cases will strengthen the point for the use of our proposed biomarkers and will better define their usefulness in the clinical field.

## 4. Materials and Methods

### 4.1. Study Design

We performed a single-blinded, monocentric, interventional, randomized study enrolling patients undergoing abdominal surgery, principally colorectal. The study was approved by the Ethical Committee of Milan Area A on 26 October 2017 (protocol number 34978/2017). Patients were recruited the day before surgery after the signature of the informed consent letter according to the Ethical Committee requests.

### 4.2. Inclusion/Exclusion Criteria

Patients undergoing elective abdominal surgery at the San Paolo University Hospital of Milan from November 2017 to April 2018 were included. The exclusion criteria were: age <18 years; pregnancy; respiratory disease with preoperative PaO_2_ < 150 mmHg; severe dyslipidemia; leukopenia (white blood cells < 2500); immunosuppression; cardioaspirin or vitamin therapy; chemotherapy; surgery with operative times <90 min; need of blood transfusion during surgery.

### 4.3. Randomization

Randomization occurred in the operating room before the intubation. The enrolled patients were randomized to receive during mechanical ventilation FiO_2_ = 0.40 versus FiO_2_ = 0.80, according to a previously written table that was blinded to the recruiters, and continued to receive the same FiO_2_ throughout the entire intraoperative period unless serious clinical problems occurred. In the postoperative period, patients enrolled in the FiO_2_ = 0.40 group or in the FiO_2_ = 0.80 group were scheduled to receive, after the awareness from general anesthesia and the extubation through oxygen mask, until 2 h after the end of surgery, O_2_ 4 L/min or O_2_ 10 L/min, respectively.

**Figure 6:**
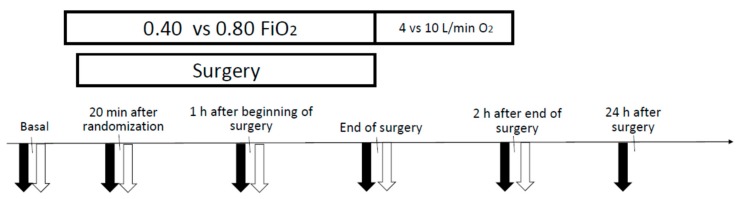
Experimental protocol. Clinical data and blood samples were collected, at the time points shown on the timeline, from patients undergoing abdominal surgery. After intubation, mechanically ventilated patients were randomized to receive FiO_2_ 0.40 versus 0.80 throughout the entire intraoperative period. After the extubation until 2 h after surgery, patients received oxygen at 4 (0.4 FiO_2_) or 10 (0.80 FiO_2_) L/min, respectively, through oxygen masks. Full arrows: arterial blood samples gathered for blood gas analysis. Empty arrows: venous blood samples gathered for oxidative stress markers.

Measurements of heart rate, blood pressure, peripheral O_2_ saturation, and the collection of arterial and venous blood samples were made at six timings, as shown in the schematic study overview of Figure 6: during the intraoperative period at the intubation (basal); twenty minutes after randomization (*t*1); 1 h after the beginning of surgery (*t*2); at the end of surgery (*t*3); during the postoperative period 2 h after the end of surgery (*t*4); and 24 h after surgery (*t*5).

### 4.4. Blood Gas Analysis

Arterial and venous gas samples were collected and immediately analyzed by a blood gas analyzer (Siemens RAPIDPoint 405, Siemens Healthcare GmbH, Erlangen, Germany) to obtain arterial and venous oxygen partial pressure (paO_2_) and arterial and venous carbon dioxide partial pressure (paCO_2_).

The arterial concentration of oxygen (CaO_2_) was calculated using the following formula:

CaO_2_ = 1.36 × Hb × SaO_2_/100 + 0.0031 × PaO_2_ (1)

where Hb = hemoglobin and SaO_2_ = arterial oxygen saturation.

### 4.5. Oxidative Stress Markers

Venous blood samples were centrifuged (4500× *g*, 5 min) immediately after they were drawn, separating plasma and red blood cells (RBCs), and frozen at −20 °C for subsequent analyses.

Plasma samples were analyzed for: 

Hydroperoxides (dROMs test, Diacron Inc., Grosseto, Italy [24]); 

Malondialdehyde (MDA) (TBARS test [31], Cell Biolabs Inc., San Diego, CA, USA);

Antioxidant barrier strength (OXY-Adsorbent test, Diacron Inc., Grosseto, Italy);

Nitrates and nitrites (NO_x_) (Griess reaction [32] after deproteinization with acetonitrile and vanadyl reduction of nitrate. Reagents from Sigma-Aldrich, Milan, Italy).

In the OXY-Adsorbent assay, a sample of plasma is treated with a physiologically occurring oxidant (hypochlorous acid) under standardized conditions of concentration and reaction time (10 min). The amount of oxidant is calculated so as to be in excess with respect to the “antioxidant capacity” of the sample to be tested. Hypochlorous acid reacts with, and mostly oxidizes, several organic substrates, and faster than those that compose the plasmatic antioxidant barrier. At the end of the reaction time, some hypochlorous acid remains unreacted, not “adsorbed” by the now completely oxidized plasma barrier. The excess oxidant, which is larger the lower the antioxidant barrier of the sample, is measured by reaction with an added chemical chromogen (*N*,*N*-diethyl-paraphenylenediamine) that can be quantified photometrically. The intra-series coefficient of variation (CV) evaluated on 20 rates of fresh serum, was equal to 2.2%, while the between-series CV on 20 aliquots of frozen serum was 6.3% [33].

Every plasma sample analysis was done in duplicate and the assays were repeated if the difference between two measurements exceeded 4%.

Glutathionyl hemoglobin (HbSSG) was measured in the cold-water hemolyzates of thawed red blood cells (RBCs) by matrix-assisted laser desorption in a time-of-flight mass spectrometer (MALDI-ToF, Bruker Autoflex II MALDI m.s., Bremen, Germany), essentially adapting a published method [34]. Briefly, to improve the reproducibility of the sample measurement, all samples were run in quadruplicate depositions, obtained from the same amount of hemoglobin loading. Thus, the hemoglobin concentration of the individual 1:100 hemolyzates (150 μL in a 96-well polystyrene plate) was measured at 420 nm in an EnSight (Perkin-Elmer, Monza, Italy) spectrophotometer and compared to that of standard human hemoglobin (Sigma-Aldrich, Milano, Italy). According to their individual values (approx. 20–50 μM), samples were diluted to a constant concentration (10 μM). For the MALDI analysis, the sample (10 μL) was mixed with an equal volume of freshly prepared sinapinic acid matrix (Sigma-Aldrich, MALDI-grade brand, 30 mg/mL in 50% *v/v* acetonitrile—0.1% trifluoroacetic acid). Four one-microliter aliquots were manually spotted in adjacent circular wells of the stainless-steel plate, air-dried at room temperature, and loaded into the Bruker Autoflex III mass spectrometer for measurement.

### 4.6. Statistics

Data are expressed as mean ± standard error of the mean (SEM). We performed Student’s unpaired *t*-test to compare the two experimental groups at each time point. To compare each value from the same group to the basal value, we performed the two-way ANOVA according to Dunnett’s method. Statistical analyses were performed using GraphPad Software (GraphPad Software Inc., San Diego, CA, USA).

## 5. Conclusions

Both hypoxic and hyperoxic conditions generate oxidative stress, although likely with different biochemical mechanisms, and can cause common cellular and systemic damage [1,13]. A better knowledge of the oxidative stress induced by hyperoxia and a rational panel of biomarkers can be useful to optimize the treatment and prevention of hypoxia during surgery.

MDA, a product of lipid peroxidation, has been confirmed as a promising marker to assess the late effects of hyperoxia-related oxidative stress. Increased lipid peroxidation, which results in cell membrane damage, is a consequence of high intraoperative and perioperative FiO_2_.

Moreover, HbSSG and NO_x_ can be useful markers for further studies with a larger sample size to better investigate the meaning of their variations. In particular, pre-surgery levels of HbSSG may assist with predicting the necessity of coping with a chronic weakness of the RBC antioxidant buffer.

In conclusion, further investigations on inflammatory markers will be fundamental to evaluate the benefits raised by decreased FiO_2_, as well as studies on the role of anti-oxidant treatments to down-regulate the pro-oxidant mechanism [35,36].

## Figures and Tables

**Table 1:** Demographic and anthropometric characteristics of the study population.

Parameter	FiO_2_ = 0.40	FiO_2_ = 0.80	*p* Value
n (women)	10 (3)	10 (4)	NS
Age, years	69 ± 4	69 ± 3	NS
Height, m	1.69 ± 0.03	1.66 ± 0.03	NS
Weight, kg	70 ± 5	68 ± 4	NS
BMI, kg/m^2^	24.5 ± 1.3	24.7 ± 1.1	NS
SpO_2_ at rest, %	96.6 ± 1.3	98.5 ± 0.5	NS
Smokers, %	20	20	NS
Preoperative hemoglobin concentration, g/dL	13.5 ± 0.8	12.6 ± 0.5	NS
Surgery running time, min	179 ± 19	164 ± 12	NS
Amount of infused liquids, mL	2411 ± 313	2060 ± 200	NS
Urine output at the end of surgery, mL	315 ± 405.28	712.5 ± 467.33	NS

Data expressed as mean ± standard error of the mean (SEM). NS: *p* < 0.05, Student’s *t*-test. SpO_2_: peripheral saturation of O_2_. BMI: body mass index.

## Supplementary Materials

The following are available online at https://www.mdpi.com/1422-0067/20/21/5492/s1.

## Abbreviations

CaO_2_	Arterial concentration of oxygen
FiO_2_	Fraction of inspired oxygen
GPx	Glutathione peroxidase
GSH	Glutathione (reduced form)
GS-SG	Glutathione (oxidized form)
Hb	Hemoglobin
HbSSG	Glutathionyl hemoglobin
MDA	Malondialdehyde
NO_x_	Nitrites and nitrates
O_2_	Oxygen
PaO_2_	Arterial partial pressure of oxygen
RBCs	Red blood cells
RNS	Reactive Nitrogen Species
ROS	Reactive Oxygen Species
SpO_2_	Peripheral saturation of oxygen
