# Supplementary material for: Oxidative Stress Markers to Investigate the Effects of Hyperoxia in Anesthesia
Source: Int J Mol Sci. 2019 Nov 4;20(21):5492. doi: 10.3390/ijms20215492 (PMC6862279; doi:10.3390/ijms20215492)
Supplement: Supplementary file 1 [file ijms-20-05492-s001.pdf]

Oxidative Stress Markers to Investigate the Effects of Hyperoxia in Anesthesia

Sara Ottolenghi, Federico Maria Rubino, Giovanni Sabbatini, Silvia Coppola, Alice Veronese, Davide Chiumello, Rita Paroni.

Supplementary materials

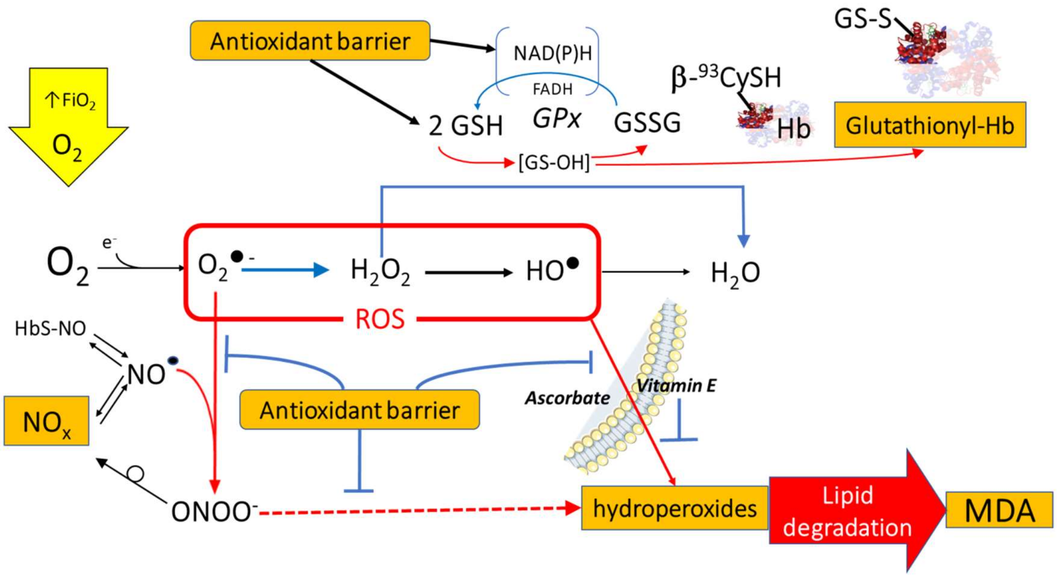

Figure S1. Some mechanisms for production of the main Reactive Oxygen Species (ROS) from excess dissolved molecular oxygen (O_2) and the bio-markers used to evaluate its effects. Excess O_2 is a source of oxidative stress, since the production of the ROS is related to its partial pressure in the relevant biological compartments. The main ROS are: superoxide (O2−•), hydrogen peroxide (H2O2) and the hydroxyl radical ($HO^•$). $O_2^{-•}$ reacts with nitric oxide ($NO^•$) to yield peroxynitrite ($ONOO^-$, an isomer of nitrate, to which it isomerizes), therefore consuming the $NO^•$ pool. The $NO^•$ pool is partially restored by $NO^•$ release from its bound forms (HbS-NO). The measurement of nitrates and nitrites (NO_x) accounts for these processes. The ROS, and especially $O_2^{-•}$ and $HO^•$, react with most organic compounds of biological structures, and especially with unsaturated plasma lipids, to yield organic **hydroperoxides**, which can be measured in plasma, and finally generate **malondialdehyde** (MDA), that can also be measured. The pool of chemically diverse, organic antioxidants constitutes the antioxidant barrier, the strength of which can be functionally evaluated. It intercepts the excess ROS, by buffering the perturbed homeostatic levels of metabolic intermediates, and regenerating compromised biological macromolecules and structures. The hub, and quantitatively prominent antioxidant process is based on the reversible conversion of glutathione (GSH) to its S-oxidized species, sulphinic acid (GS-OH) and glutathione disulfide (GS-SG), with a concomitant chemical reduction of H_2O_2 into water. Reducing enzymes, such as NADH-FADH$_2$-dependent glutathione peroxidase (GPx), in turn recycle GS-SG to GSH. In circulating erythrocytes, the produced GS-OH or GS-SG react with a cysteine residue of hemoglobin (Hb-SH) to produce an analog of glutathione disulfide, **glutathionyl-hemoglobin** (HbS-SG, that can be specifically measured), from which GSH can be liberated through processes that involve catalytic amounts of reducing thiols or by membrane-bound protein-disulfide isomerases. Orange squares: oxidative stress markers considered in this study. Blue arrows: reduction processes. Red arrows: oxidation processes.

Further comments on the physiological meaning of some indicators of oxidative stress.

The plasma NOx level may give information about the effect of oxidative stress on modulation of the vasorelaxation by NO•. In fact, high levels of ROS react with NO to yield, among others, the peroxynitrite (ONOO-) ion [1] which isomerizes to NOx. This reaction decreases the size of NO• pool.

The main antioxidant mechanism in RBC is H2O2 scavenging by reduced glutathione (GSH) and final generation of glutathionyl hemoglobin. to yield glutathione sulphinic acid (GS-OH). This short-half-life intermediate product further reacts with either GSH to yield "oxidized" glutathione disulphide (GSSG), or with the cysteine residue of the β chain of the hemoglobin, yielding the mixed-disulfide HbSSG, which acts as the main thiol reducing buffer of RBC [2]. GSSG is reduced back to GSH by the enzyme glutathione peroxidase (GPx), and its disulfide exchange reaction with HbS-SG liberates free-thiol hemoglobin, and GSSG that is reduced by GPx. This net cycle allows the ROS to be reduced into water (top right figure S1). Therefore, the observed higher HbSSG in the FiO2=0.80 group at the end of the surgery may be considered as another sign of the consumption of the GSH component of the antioxidant barrier, even if some details of the involved pathways still need investigation [3].

References

1. Kalyanaraman, B., *Teaching the basics of redox biology to medical and graduate students: Oxidants, antioxidants and disease mechanisms.* Redox Biol, 2013. **1**: p. 244-57.
2. Maria Rubino, F., et al., *Measurement of Glutathionylated Haemoglobin by MAL-DI-ToF Mass Spectrometry as a Biomarker of Oxidative Stress in Heavy Smokers and in Occupational Obese Subjects.* International Journal of Analytical Mass Spectrometry and Chromatography, 2013. **01**(01): p. 22-30.
3. Colombo, G., et al., *Cellular redox potential and hemoglobin S-glutathionylation in human and rat erythrocytes: A comparative study.* Blood Cells Mol Dis, 2010. **44**(3): p. 133-9.
